# Tobacco Smoking in Islands of the Pacific Region, 2001–2013

**DOI:** 10.5888/pcd12.150155

**Published:** 2015-12-03

**Authors:** Tara Kessaram, Jeanie McKenzie, Natalie Girin, Adam Roth, Paula Vivili, Gail Williams, Damian Hoy

**Affiliations:** Author Affiliations: Jeanie McKenzie, Natalie Girin, Adam Roth, Paula Vivili, Damian Hoy, Public Health Division, Secretariat of the Pacific Community, Noumea, New Caledonia; Gail Williams, University of Queensland, Brisbane, Queensland, Australia.

## Abstract

We provide an overview of tobacco smoking patterns in Pacific island countries and territories to facilitate monitoring progress toward the goal of a Tobacco-Free Pacific by 2025. We examined data from 4 surveys conducted in the region between 2001 and 2013, including the STEPwise approach to surveillance for adults (25–64 years); the Global School-Based Student Health Survey and the Global Youth Tobacco Survey (students 13–15 years); and the Youth Risk Behavior Surveillance System (grade 9–12 students) in United States affiliated Pacific Islands (USAPIs). Adult smoking prevalence ranged from less than 5% of women in Vanuatu to almost 75% of men in Kiribati. Smoking prevalence among students (13–15 years) ranged between 5.6% and 52.1%. There were declines in smoking among youths in many USAPIs. To achieve the tobacco-free goal and reduce disease burden, accelerated action is needed to align national legislation with international agreements and build capacity for tobacco control at all levels.

## Background

Killing almost 6 million people annually, tobacco use continues to plague global population health, especially in low-income and middle-income countries (1). The epidemic persists in the 22 Pacific island countries and territories (PICTs) where tobacco use is a leading risk factor for disease (2). Fundamentally, tobacco use endangers the sustainable development of these nations and threatens their collective vision of healthy islands (3).

Since 1947, the 22 PICTs have collaborated for development and improvement in public health through the Secretariat of the Pacific Community. Recognizing the public health gains of curtailing tobacco use and the crisis of noncommunicable diseases (NCDs), ministers of health have committed to achieving a Tobacco-Free Pacific by 2025 (4).

To advance and evaluate tobacco control efforts in the region, ongoing surveillance of tobacco use is required. For PICTs, the most recent review of smoking specifically was undertaken in 2007 (5). Since then, several PICTs have published results from standardized surveys of tobacco smoking (6–11). By collating the new wealth of data, we provide an updated, comprehensive assessment of adult and youth smoking patterns in the Pacific. This analysis will guide national and regional initiatives for tobacco control. Furthermore, it will provide a revised baseline from which to measure and monitor the extent and equity of the region’s progress toward becoming tobacco-free.

## Methods

Surveys used to assess national patterns of tobacco use in PICTs include the STEPwise approach to surveillance (STEPS); the Global School-Based Student Health Survey (GSHS); the Global Youth Tobacco Survey (GYTS); and in the US Affiliated Pacific Islands (USAPIs), the Youth Risk Behavior Surveillance System (YRBSS) and the Behavioral Risk Factor Surveillance System.

Because of its implementation in several PICTs, we selected STEPS as the primary data source on adult smoking. We present sex-specific data from 15 PICTs on current smoking, from 14 PICTs on daily smoking, and from 7 PICTs on use of manufactured cigarettes by daily smokers. STEPS usually uses a multistage cluster design with probability proportional to size sampling (12). Two smaller PICTs — Niue and Tokelau — designed STEPS to survey all members of the target adult population. STEPS collects information on tobacco use through a face-to-face interviewer-administered questionnaire. Sample, nonresponse, and population weights are applied to the data. This standardized methodology enables valid intercountry comparisons across PICTs that have published full survey reports (6). We also included the survey conducted in Wallis and Futuna that was based on STEPS methodology (7), and New Caledonia’s Baromètre Santé (8), which like STEPS used a multistage sampling design and in-person interviewing.

Most STEPS reports provided data on adult smoking prevalence in 10-year age brackets; French Polynesia reported in 20-year age brackets. Accordingly, we standardized the age and sex-specific prevalence of current smoking (usually defined as smoking within the previous 12 months), daily smoking (usually defined as smoking any tobacco product every day), and percentage of daily smokers using manufactured cigarettes to the World Health Organization (WHO) world population, producing a summary rate for those aged 25 to 64 years (13). Before standardization, we developed and compared 2 Pacific reference populations with the WHO standard population. Finding minimal difference between the reference populations and the WHO standard population, we adopted the WHO population. We calculated standard errors and confidence intervals for the age-standardized estimates of prevalence using the methods described by Breslow and Day (14).

For youths, we selected GYTS and GSHS, because these standardized surveys enable intercountry comparisons. Both use similar methodologies and provide results for those aged 13 to 15 years old. GYTS uses a multistage sample design in which schools are selected proportional to enrollment size, and classes are selected randomly (9). GSHS often uses similar 2-stage sampling of schools and classes and obtains information on tobacco use through a self-administered anonymous questionnaire (15). Results from both surveys are weighted as required.

For GYTS, we extracted data from the Global Tobacco Surveillance System database or from fact sheets published by the Centers for Disease Control and Prevention (CDC) (9). For GSHS, we used fact sheets available through WHO (10). Both surveys defined current cigarette smoking as smoking cigarettes on at least 1 day within the previous 30 days. GYTS asked about manufactured cigarettes and hand-rolled cigarettes; GSHS did not specify cigarette type within the corresponding question. Conscious of this difference, we present the most recently available sex-specific estimate for current smoking prevalence among students in 18 PICTs. We also describe age at initiation of students who ever smoked cigarettes, the percentage of student smokers who tried their first cigarette before age 14 years (GSHS) for 7 PICTs, and the percentage who now desire to quit smoking (GYTS) for 10 PICTs, by sex.

YRBSS surveys students in grades 9 through 12 and uses methodology similar to GSHS (16). YRBSS has been repeated at least twice in 5 of the 6 USAPIs (American Samoa, Guam, Commonwealth of the Northern Mariana Islands [CNMI], the Republic of the Marshall Islands [RMI], and Palau). We extracted data from Youth Online, the CDC database of YRBSS results, which enables statistical testing between survey years (11). We used this function for results from Guam, because the other USAPIs designed YRBSS to include all members of the target student population. We present sex-specific trends in the prevalence of previous 30-day smoking of cigarettes (type not specified), from 2001 through 2013 among students in grades 9 through 12.

The 4 surveys provided information on tobacco use by adults or youths across 21 PICTs (Table 1). Comparable data were unavailable for the Pitcairn Islands.

**Table 1:** STEPwise Approach to Surveillance (STEPS), Global School-Based Student Health Survey (GSHS), Global Youth Tobacco Survey (GYTS), and Youth Risk Behavior Surveillance System (YRBSS) Surveys Used to Assess National Patterns of Tobacco Use, by Year and Pacific Island Country or Territory^a^

Country or Territory	STEPS^b^	GSHS	GYTS	YRBSS
American Samoa	2004		2005	2007, 2011
Commonwealth of the Northern Mariana Islands			2001, 2004^c^	2003, 2005, 2007
Cook Islands	2003–2004	2011	2003, 2008	
Federated States of Micronesia	2002 (Pohnpei), 2006 (Chuuk)		2007	
Fiji	2002	2010	2005, 2009	
French Polynesia	2010			
Guam			2002, 2011	2001, 2007, 2011, 2013
Kiribati	2004–2006	2011	2009	
New Caledonia	2010		2010	
Nauru	2004	2011		
Niue	2011	2010		
Palau			2000, 2005	2001, 2003, 2005, 2007, 2009, 2011
Papua New Guinea	2007–2008		2007	
Republic of the Marshall Islands	2002		2009	2003, 2007, 2009
Samoa	2002	2011	2007	
Solomon Islands	2005–2006	2011	2008	
Tokelau	2005			
Tonga	2004	2010	2010	
Tuvalu		2013	2006	
Vanuatu	2011	2011	2007	
Wallis and Futuna	2009			

a No comparable data were available for Pitcairn Islands.

b The New Caledonia survey used a methodology similar to STEPS. Age-specific data for adults by age group in Papua New Guinea and Samoa were not available. The survey for Wallis and Futuna was based on STEPS methodology.

c Youth Tobacco Survey, which uses the same methods as GYTS, was conducted in 2004.

## Results

### Adults (25–64 years)

Although age-standardized prevalence of current smoking varied widely (Table 2), overall it was high among men; at least one-quarter smoked in Niue and almost three-quarters smoked in Kiribati. There was a wider range of prevalence among women, with minimal smoking in Vanuatu (<5%) to the highest prevalences of current smoking in Tokelau (59.3%) and Nauru (56.1%; 95% CI, 52.7–59.6). Approximately 20% or more of women were current smokers in 11 PICT populations. In all PICTs, apart from Nauru and French Polynesia, point prevalence of smoking was higher among men than women.

**Table 2:** Age-Standardized Prevalence of Current Tobacco Smoking and Daily Tobacco Smoking Among Those Aged 25 to 64 Years and Use of Manufactured Cigarettes by Daily Smokers, by Sex and Pacific Island Country or Territory^a^

Country or Territory	Current Smokers^b^		Daily Smokers		% Daily Smokers Using Manufactured Cigarettes	
	Men, % (95% CI)	Women, % (95% CI)	Men, % (95% CI)	Women, % (95% CI)	Men, % (95% CI)	Women, % (95% CI)
**American Samoa**	48.4 (44.1–52.7)	29.1 (25.2–33.1)	37.7 (34.4–40.9)	21.1 (18.9–23.3)	NA	
**Cook Islands**	45.3 (42.2–48.3)	38.7 (35.4–42.1)	36.6 (33.1–40.1)	27.3 (24.4–30.1)	52.0 (42.8–61.2)	69.5 (59.9–79.0)
**Federated States of Micronesia**						
Chuuk	50.4 (47.0–53.9)	14.1 (11.6–16.6)	46.6 (42.9–50.3)	10.8 (8.5–13.1)	84.8 (81.0–88.6)	81.6 (74.3–89.0)
Pohnpei	40.2 (35.9–44.4)	21.7 (18.9–24.4)	33.4 (29.8–37.0)	16.4 (14.2–18.6)	90.3 (85.6–95.0)	84.9 (79.0–90.9)
**Fiji**	59.7	19.6	29.2 (25.8–32.6)	4.6 (3.4–5.8)	NA	
**French Polynesia**	36.5 (33.2–39.8)	41.4 (38.3–44.5)	NA		NA	
**Kiribati**	74.7 (70.4–79.1)	49.4 (45.2–53.6)	73.0 (68.7–77.4)	46.4 (42.4–50.4)	43.2 (38.0–48.4)	45.2 (39.9–50.5)
**New Caledonia**	50.4 (46.9–53.8)	40.2 (37.5–42.8)	48.7 (45.3–52.2)	39.1 (36.5–41.7)	NA	
**Nauru**	47.9 (44.2–51.5)	56.1 (52.7–59.6)	44.4 (40.8–48.0)	52.0 (48.5–55.5)	NA	
**Niue^c^**	25.4	16.1	17.1	9.8	91.3	93.9
**Republic of the Marshall Islands**	36.7 (32.7–40.7)	6.8 (5.6–8.0)	33.2 (29.1–37.3)	5.2 (4.1–6.3)	NA	
**Solomon Islands**	52.6 (48.2–57.0)	24.3 (21.0–27.6)	42.8 (38.7–46.9)	16.5 (13.9–19.2)	57.4 (49.5–65.2)	52.6 (44.1–61.0)
**Tokelau^c^^,^ d**	62.4	59.3	54.0	49.0	NA	
**Tonga**	46.5 (41.8–51.3)	14.0 (8.6–19.3)	41.8 (36.3–47.3)	11.9 (7.9–16.0)	82.9 (76.5–89.3)	79.0 (67.9–90.2)
**Vanuatu**	43.9 (41.5–46.3)	3.6 (2.7–4.6)	23.1 (20.9–25.3)	1.4 (0.9–1.9)	47.5 (41.7–53.2)	69.4 (59.4–79.5)
**Wallis and Futuna**	68.6 (61.4–75.9)	32.1 (26.1–38.2)	61.9 (54.3–69.5)	27.0 (21.2–32.8)	NA	

Abbreviations: CI: confidence interval; NA, not available.

a Sources: STEPwise approach to surveillance and similar surveys (in Wallis and Futuna and in New Caledonia) conducted between 2002 and 2011.

b Results for current smoking are from Kessaram et al (17). Current smoking was defined as smoking tobacco within the previous 12 months for all surveys, except French Polynesia, Nauru, and New Caledonia, for which a timeframe was not specified. We estimated the prevalence of current smoking for Fiji by adding daily and nondaily smoking prevalence; confidence intervals were therefore not calculated.

c Confidence intervals were not applicable to results because the surveys were designed to include all members of the target adult population.

d Because of a misprint in the reported age-specific rates of current and daily smoking in women in the Tokelau report, we recalculated these using the case numbers provided and the age-specific sample numbers listed elsewhere in the report and verified by the World Health Organization.

A higher percentage of men than women smoked daily in all PICT populations, except for Nauru. Among men in 8 populations, prevalence exceeded 40%. Although there was wide variability among women, in 9 PICT populations more than 15% smoked daily. In several PICTs, the prevalence of daily smoking was nearly as high as current smoking (Table 2).

In 4 PICT populations, around 80% of male and female daily smokers used manufactured cigarettes. Among women, use was lowest in the Solomon Islands and Kiribati. Less than 60% of male daily smokers used manufactured cigarettes in these PICTs, as well as in Vanuatu and the Cook Islands (Table 2).

### Youths (13–15 years)

**Figure 1:**
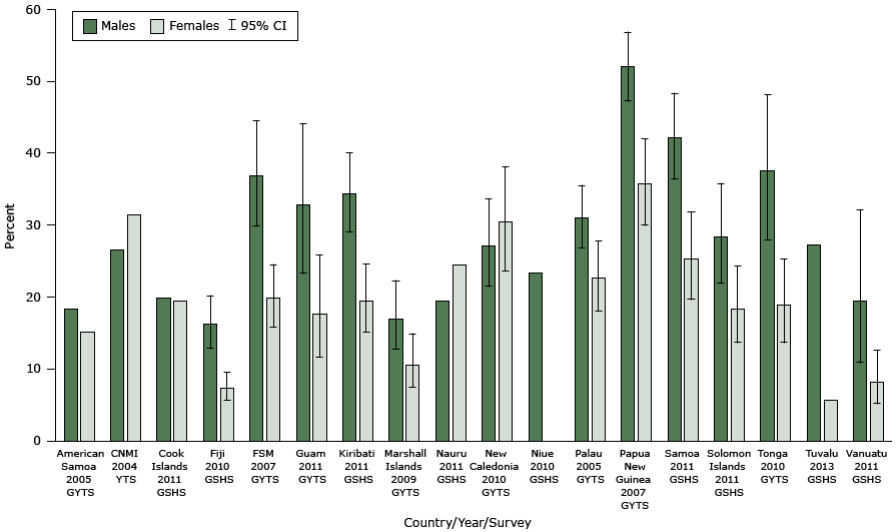
Prevalence of current smokers among students aged 13 to 15 years, by sex and Pacific island country or territory. Abbreviations: CI, confidence interval; CNMI, Commonwealth of the Northern Mariana Islands; FSM, Federated States of Micronesia. Sources: Global Youth Tobacco Surveys (GYTS), Youth Tobacco Survey (YTS), and Global School-Based Student Health Surveys (GSHS) conducted between 2004 and 2013. Current smoking was defined in the surveys as smoking cigarettes on at least 1 day within the previous 30 days. GYTS included manufactured cigarettes and hand rolled-cigarettes. Type of cigarette was not specified in the corresponding question in the GSHS. Prevalence among females was not reported for Niue (sample size less than 20 students). GYTS and GSHS were both undertaken in Tonga in 2010; the GYTS results are presented in the figure, and the GSHS found that 19.2% (95% CI, 15.8–23.0) of male students and 23.8% (95% CI, 20.3–27.7) of female students smoked cigarettes in the previous 30 days. Equivalent data were unavailable for French Polynesia, the Pitcairn Islands, Tokelau, and Wallis and Futuna. CIs were not applicable to the American Samoa, CNMI, Cook Islands, Niue, and Nauru results because the surveys were designed to include all members of the target student population. CIs were not reported for Tuvalu. **Table:** 

Smoking prevalence among students ranged from 5.6% to 52.1%. As measured by GYTS or GSHS, over 25% of male students in 11 PICTs smoked cigarettes within the previous 30 days (Figure 1). In 13 PICTs, over 15% of female students were current smokers. Smoking prevalence was similar between the sexes in the Cook Islands; in CNMI, Nauru, and New Caledonia, point prevalence was higher for females.

Country/Year/Survey	Males, % (95% CI)	Females, % (95% CI)
American Samoa 2005 GYTS	18.3	15.1
CNMI 2004 YTS	26.6	31.5
Cook Islands 2011 GSHS	19.9	19.4
Fiji 2010 GSHS	16.2 (12.9–20.2)	7.4 (5.7–9.5)
FSM 2007 GYTS	36.9 (29.9–44.5)	19.8 (15.9–24.5)
Guam 2011 GYTS	32.9 (23.3–44.1)	17.7 (11.7–25.8)
Kiribati 2011 GSHS	34.3 (29.1–40.0)	19.5 (15.2–24.6)
Marshall Islands 2009 GYTS	17.0 (12.8–22.3)	10.6 (7.5–14.9)
Nauru 2011 GSHS	19.5	24.5
New Caledonia 2010 GYTS	27.1 (21.5–33.6)	30.5 (23.7–38.1)
Niue 2010 GSHS	23.3	
Palau 2005 GYTS	31.0 (26.9–35.5)	22.6 (18.1–27.8)
Papua New Guinea 2007 GYTS	52.1 (47.3–56.8)	35.8 (30.0–42.0)
Samoa 2011 GSHS	42.2 (36.4–48.3)	25.3 (19.7–31.8)
Solomon Islands 2011 GSHS	28.3 (21.9–35.8)	18.4 (13.7–24.3)
Tonga 2010 GYTS	37.5 (28–48.1)	18.9 (13.7–25.3)
Tuvalu 2013 GSHS	27.2	5.6
Vanuatu 2011 GSHS	19.4 (10.9–32.1)	8.2 (5.2–12.6)

In 7 PICTs for which GSHS data on age at initiation were available, over half of students who had ever smoked cigarettes first tried one before age 14; in Nauru and the Cook Islands, over 90% of males and females who had ever smoked did so before age 14 years. Within PICT surveys reporting on the desire to stop smoking now (GYTS), apart from males in New Caledonia, most smokers wanted to quit (Table 3).

**Table 3:** Percentage of Students Aged 13 to 15 Years Who Had Ever Smoked and Who First Tried a Cigarette Before Age 14 Years (Global School-Based Student Health Survey) and Percentage of Current Smokers Aged 13 to 15 Years Who Want to Quit Now (Global Youth Tobacco Survey), by Sex and Pacific Island Country or Territory

Country or Territory	Desire to Quit Smoking (Global Youth Tobacco Survey^a^)			Tried Cigarettes Before 14 Years (Global School-Based Student Health Survey^b^)		
	Survey Year	Males, % (95% CI)	Females, % (95% CI)	Survey Year	Males, % (95% CI)	Females, % (95% CI)
Cook Islands^c^	2008	88.1 (84.8–90.8)	71.1 (67.4–74.5)	2011	90.4	91.3
Federated States of Micronesia	2007	86.4 (78.8–91.6)	91.7 (85.1–95.5)	NA		
Fiji	2009	84.8 (68.3–93.6)	71.2 (49.0–86.5)	2010	72.8 (61.5–81.8)	65.2 (54.7–74.4)
Kiribati	2009	83.7 (74.6–90.0)	93.5 (81.4–97.9)	2011	78.9 (74.4–82.7)	72.7 (60.4–82.3)
Nauru^c^				2011	93.0	90.9
New Caledonia	2010	38.0 (21.5–57.9)	66.5 (53.6–77.4)	NA		
Palau 2005	2005	73.5 (64.0–81.2)	88.0 (76.6–94.3)	NA		
Papua New Guinea	2007	82.6 (75.8–87.9)	81.4 (75.5–86.1)	NA		
Samoa				2011	89.0 (85.2–91.9)	83.0 (76.9–87.8)
Solomon Islands	2008	95.9 (81.4–99.2)	85.4 (73.1–92.6)	2011	65.1 (55.5–73.7)	56.1 (43.1–68.4)
Tonga	2010	83.0 (75.4–88.7)	78.8 (58.5–90.8)	2010	82.1 (77.4–86.0)	80.5 (75.5–84.7)
Vanuatu	2007	83.8 (79.7–87.3)	85.4 (79.9–89.6)	NA		

Abbreviation: CI: confidence interval; NA, not available.

a Survey question: “Do you want to stop smoking now?”

b Survey question: “How old were you when you first tried a cigarette?”

c CIs are not applicable to Global School-Based Student Health Survey results because the surveys were designed to include all members of the target population.

### Youth trends (grades 9–12)

**Figure 2:**
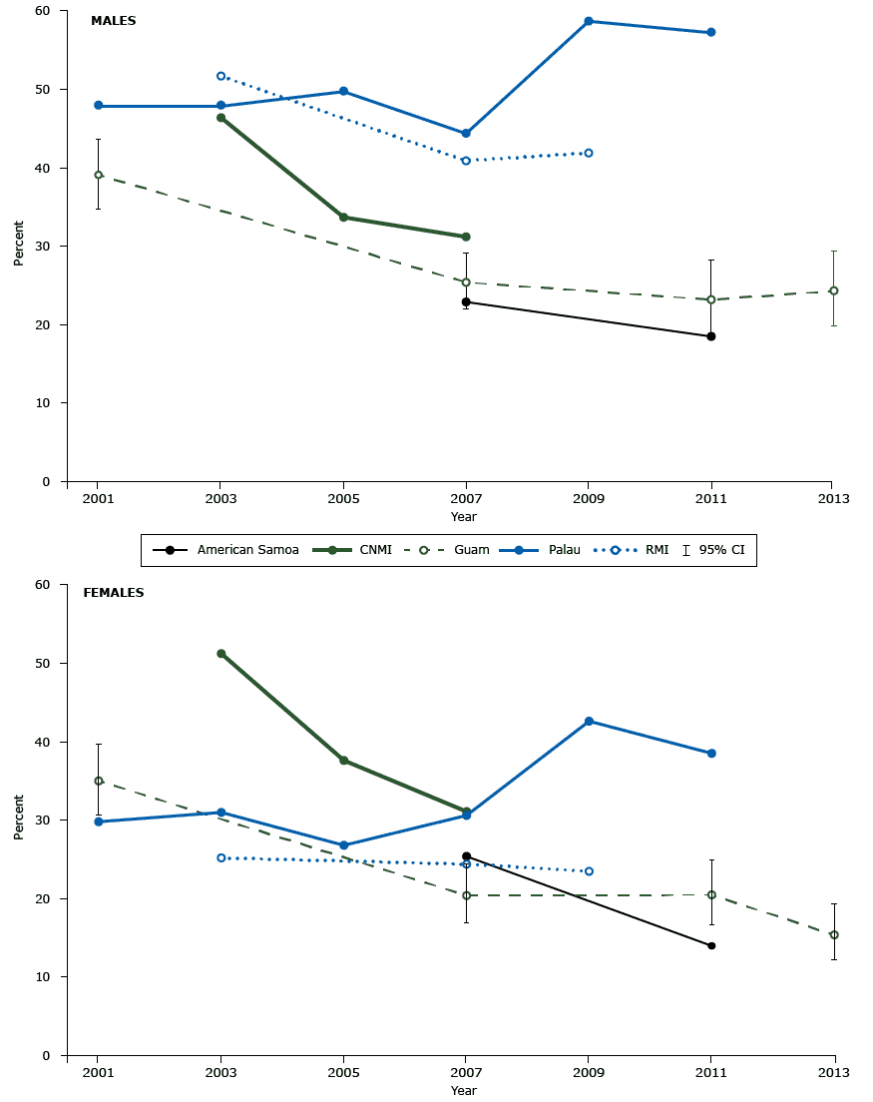
Prevalence of current smokers among male and female students in grades 9 to 12, by survey year and Pacific island country or territory. Abbreviations: CI, confidence interval; CNMI, Commonwealth of the Northern Mariana Islands; RMI, Republic of the Marshall Islands. Source: Youth Risk Behavior Surveillance System [YRBSS], 2001 to 2013. YRBSS results for CNMI for 2007 were obtained from Lippe et al (18). YRBSS results for RMI for 2009 were obtained from Substance Abuse Epidemiological Profile 2010 (19). Current smoking was defined as having smoked cigarettes on at least 1 day within the previous 30 days. For Guam only, 95% confidence intervals are presented because surveys in American Samoa, CNMI, Palau, and RMI were designed to include all members of the target student population. **Table:** 

In American Samoa, CNMI, RMI, and Guam, the YRBSS found overall declines in smoking cigarettes in the previous 30 days by males and females in grades 9 to 12 (Figure 2). In RMI, however, prevalence was similar among males in 2007 and 2009, and the decline in smoking by females was slight. Of the most recent estimates for the USAPIs, smoking prevalence was highest for both sexes in Palau, where prevalence increased overall between 2001 and 2011. In Guam, changes in smoking prevalence between 2007 and 2013 were not significant for either sex.

Sex/Year	American Samoa	CNMI	Guam (95% Confidence Interval)	Palau	RMI
**Males**					
2001			39.1 (34.8–43.6)	48.0	
2003		46.4		48.0	51.7
2005		33.7		49.8	
2007	22.9	31.2	25.4 (22.0–29.1)	44.4	40.9
2009				58.7	41.9
2011	18.5		23.2 (18.9–28.2)	57.3	
2013			24.3 (19.9–25.4)		
**Females**					
2001			35.0 (30.7–39.7)	29.8	
2003		51.2		31.0	25.2
2005		37.6		26.8	
2007	25.4	31.1	20.4 (16.9–24.4)	30.6	24.4
2009				42.6	23.5
2011	14.0		20.5 (16.7–24.9)	38.5	
2013			15.4 (12.2–19.3)

## Discussion

This article reveals a wide range in adult and youth smoking prevalence across the Pacific region, with generally higher levels among males than females. The addictive nature of smoking is reflected in many PICTs, with similar adult rates of current and daily smoking. Young people start experimenting with cigarettes early on; having become smokers, they then wish to quit. Smoking declined in some USAPIs, although this finding is not consistent between islands or equal between the sexes.

WHO estimates indicate that within the Western Pacific region, prevalence of current tobacco smoking is lowest for males in Australia (21%) and New Zealand (21%) and is under 5% for females in many Asian countries (1); in comparison, Pacific countries have some of the highest prevalences of current tobacco smoking (1). Our results support this evidence, demonstrating alarming levels especially among men. High prevalence among women must not be overlooked, particularly when prevalence of daily tobacco smoking approaches or exceeds prevalence among men, as observed in Nauru and in Austria and Sweden (1). Potentially reflecting changing social norms and tobacco marketing tactics, these results may be a harbinger of future increases in tobacco use among women. We found that current smoking prevalence was higher than daily smoking; the degree of difference varied within the region, however, and may reflect variations in tobacco availability or affordability.

A collation of GSHS data published as of March 2010 (20) reported that the highest current smoking prevalence among male students was in Zamora, Ecuador (32.7%), and among females in a metro region of Chile (38.1%). In comparison, smoking prevalence was higher for male students in several PICTs and generally lower for female students. Although the percentage of youths wishing to quit is encouraging, this finding indicates the need for cessation support.

Declines in smoking such as that recorded by YRBSS in Guam are encouraging. Researchers attributed this trend, also observed for adults, to comprehensive and collaborative tobacco control policy in Guam, particularly legislative measures (21). The absence of marked reductions across PICTs, however, indicates present and future disparities in tobacco-related disease. Qualitative research on underlying drivers of tobacco use would help explain observed trends.

## Limitations

The surveys we used were conducted at various points during the past 15 years; patterns of smoking may have changed since the survey. Age-specific data for adults were not available for Samoa and Papua New Guinea (PNG). This work therefore lacks information for a substantial proportion of the adult Pacific population. For some STEPS surveys, sampling frames were restricted because of cost and logistical challenges. Therefore, results may not be nationally representative. Additionally, the youth surveys represent only those attending school.

We focused mainly on those aged 25 to 64 years and those aged 13 to 15 years. Data for those aged 15 to 24 years were not available for all PICTs through STEPS, although this demographic group was partially captured in YRBSS. For many PICTs, however, information on smoking by preteenagers, adolescents, and young adults is limited. Further, we examined only tobacco smoking and cigarette use. Tobacco chewing, often concomitantly with chewing betel nut, is common, particularly in parts of Melanesia and Micronesia (22). Finally, although manufactured cigarettes are often smoked, increasing use of locally grown tobacco is a serious concern; data on this use are needed.

## Implications for Policy

The Tobacco-Free Pacific goal calls for tobacco use to be reduced to below 5% among adults in each PICT (4). Achieving this goal will present varying degrees of challenge to different islands. Tobacco control efforts must redress the environmental influences contributing to the early initiation of smoking and must respond to the differences in smoking between men and women.

The WHO Framework Convention on Tobacco Control (FCTC) (23) and the corresponding “best buy” approaches for tobacco in the context of NCDs (24) require accelerated implementation to reduce the social, health, and economic burden of tobacco use in PICTs. All 14 Pacific WHO member countries have signed the FCTC. Because of nonratification by the United States, Guam, American Samoa, and CNMI are excluded from the FCTC. New Caledonia, Wallis and Futuna, and French Polynesia are also not signatories. Although some of these PICTs are nevertheless active in tobacco control, exclusion from the FCTC creates inequalities within the Pacific and necessitates reliance on voluntary implementation of the treaty’s provisions (25).

Substantial advances are being made in the Pacific, although the degree to which legislation is “FCTC compliant” varies. All countries tax tobacco, although levels and aims may differ; the Cook Islands, Federated States of Micronesia (FSM), and Samoa connect tax and price policies to health objectives (26). The Cook Islands, PNG, RMI, and Samoa implemented a comprehensive ban on tobacco advertising, promotion, and sponsorship within 5 years of entry into the FCTC (26). RMI and the Cook Islands have met obligations to adopt and implement measures that protect people from exposure to tobacco smoke in indoor public places, workplaces, transport, and other public spaces as appropriate. This FCTC requirement has been partially met in FSM, Fiji, Palau, PNG, Samoa, and Solomon Islands (26). There are concerns, however, regarding enforcement of and exemptions to these policies (27). Tobacco industry interference in PICTs has also been documented (28), despite FCTC requirements to protect policy from industry interests. Enforcement of legislation can be constrained by limited funds and capacity.

PICTs and development partners continue to collaborate on these challenges. There are substantial opportunities ahead. These include 1) using tax and price mechanisms to reduce demand, and where possible, using revenue to provide sustainable funding for tobacco control; 2) regulating the packaging of tobacco products, including requiring extensive written and pictorial health warnings; 3) establishing and enforcing smoke-free environments, including in public places; 4) forming multisectoral coordination mechanisms to develop and monitor progress on national tobacco action plans; 5) augmenting civil society involvement; 6) providing cessation support services; and 7) revising local tobacco legislation and policy according to FCTC obligations and evolving best practices (1,23).

Initiatives can build on factors known to further tobacco control in the Pacific. These include strong political, government, and community leadership to drive the agenda and partnerships such as Pacific Partners for Tobacco Free Islands (25). Capacity building and engagement with local champions, traditional leaders, faith-based organizations, and antitobacco advocacy organizations are essential to implementing effective policies (21,25,27).

## Conclusion

The evidence presented on smoking prevalence demonstrates the need for urgent action in the Pacific. Evaluating the impact of such action and monitoring progress toward the regional goal is critical. Surveillance systems in the Pacific must be strengthened, with all PICTs undertaking repeated, standardized, representative surveys.

To reduce tobacco use and eliminate inequalities, tobacco control initiatives need to be comprehensive and adequately resourced. This requires strengthened political commitment, involvement of multiple sectors, and cross-government approaches. Protecting public health policy from tobacco industry interference and monitoring industry activity will be crucial for success. The commitment required by PICTs to achieve a Tobacco-Free Pacific cannot be underestimated.
